# The relationship between social frailty and cognitive impairment among older adults: the role of various types of internet use

**DOI:** 10.3389/fpubh.2024.1424465

**Published:** 2024-09-06

**Authors:** Kyungwon Choi, Young Ko

**Affiliations:** ^1^Department of Nursing, Korea National University of Transportation, Chungbuk, Republic of Korea; ^2^College of Nursing, Gachon University, Incheon, Republic of Korea

**Keywords:** social frailty, cognitive function, internet use, older adults, community

## Abstract

**Introduction:**

This study aimed to explore the role of internet use in the relationship between social frailty and cognitive function among Korean older adults.

**Methods:**

A nationally representative survey of community-dwelling older adults in Korea was used in the analysis (*N* = 8,639).

**Results:**

All types of internet use were significantly associated with cognitive impairment and played a significant role in the relationship between social frailty and cognitive impairment. The advantage of internet use for information searching (OR 0.40, 95% CI 0.35–0.46) was the greatest for cognitive function, followed by internet use for instrumental use (OR 0.59, 95% CI 0.53–0.66). Internet use for entertainment exhibited the greatest influence in the relationship between social frailty and cognitive impairment, with interpersonal communication ranking second in significance. Internet use regulates the relationship between social frailty and cognitive impairment in older adults. The influences of internet use vary depending on the type of online activity and the levels of social frailty.

**Discussion:**

This highlights the importance of considering various forms of internet use when developing non-pharmacological interventions to mitigate the impact of social frailty on cognitive decline.

## 1 Introduction

With the aging population, maintaining cognitive health has become crucial for the well-being and quality of life of older adults, as it significantly impacts their ability to live independently (1) and is thus important for preventing functional limitation and disability in later life (2). Age-related cognitive decline can manifest as a decline in anti-interference ability, memory, and reaction time, among other symptoms (3). Cognitive decline can directly impact the daily functioning of older individuals, including tasks such as cooking, financial management, medical treatment, and other activities (4). Currently, there is a general trend to delay cognitive decline through effective non-pharmacological interventions (5). Therefore, studies to identify modifiable risk factors for cognitive decline have become increasingly important.

Frailty is a widely used concept that describes a state of vulnerability associated with multi-system decline in physiological reserve and diminished stress resistance, resulting in increased risk of adverse outcomes including disability, hospitalization, and death (6, 7, 8). Frailty was originally defined primarily as a physical condition, but is now recognized as a multidimensional condition encompassing cognitive and social aspects as well (9). Among them, physical frailty and cognitive frailty have been extensively documented. Studies on social frailty, however, are relatively limited. Social frailty is a concept that reflects a spectrum of social functions and has gained attention recently. It can be defined as a state of being at risk of losing or having lost social resources, social behaviors or activities, and self-management abilities needed to fulfill basic social needs (10). Social factors play a significant role in maintaining physical, cognitive, and mental functioning, as well as the ability to perform Instrumental Activities of Daily Living (IADL) in older adults. Recent studies have shown that social frailty is a strong predictor of functional impairment, depression, cognitive decline, and mortality among community-dwelling older adults (11, 12, 13, 14, 15, 16). Social activities usually require higher degrees of skill. Diminished social activity, a key component of social frailty, is linked to a faster rate of motor function decline in older adults and an increased risk of cognitive decline (11, 12, 13, 17, 18), whereas a larger social network has a protective effect against dementia (19). The cognitive reserve hypothesis suggests that cognitive stimulation augments an individual’s reserve capacity, which is believed to be instrumental in maintaining cognitive function with age (20). Adopting a less engaging lifestyle throughout life may accelerate the loss of cognitive function due to lower cognitive reserve (21). Maintaining an active social network and participating in social activities are important cognitively challenging activities that, as such, provide cognitive stimulation and contribute to the promotion of cognitive reserve (22, 23). Therefore, older adults with social frailty may experience cognitive impairments. However, few studies have examined social frailty and its association with cognitive impairments.

Along with rapid advances in information and communication technology, internet usage has become an integral part of older adults’ daily lives, who have progressively transitioned from offline to online activities. Previous studies have shown that internet use has significant effects on the health status of older adults and helps reduce social isolation. Internet usage has been found to have an effect on cognitive functioning. Empirical research has shown that internet use improves cognitive performance (5, 24) or mitigates the overall cognitive decline in old age (25, 26). Furthermore, the internet can eliminate the physical barriers caused by age-related reductions in mobility, enabling older adults to maintain existing social networks and even form new ones (27). Moreover, it can also be an important means for increasing social capital by facilitating greater information accessibility and sustaining social connections (28). Based on the above studies, it is plausible that internet usage among older adults with social frailty could promote the expansion of their social connections, thereby serving as a cognitive stimulant and potentially delaying the advancement of cognitive impairments. However, few studies have explored the underlying mechanism of this relationship. Therefore, this study aims to explore the role of internet use between social frailty and cognitive function.

## 2 Materials and methods

### 2.1 Study design

This was a cross-sectional study with secondary data from the 2020 National Survey of Older Koreans (NSOK) and was officially approved by the Institutional Review Board of the Korea Institute for Health and Social Affairs (KIHSA IRB Number: 2020–36) (29).

### 2.2 Data and participants

The 2020 LPOPS involved a sample of community-dwelling older adults aged ≥65 years residing in 17 regions in both urban and rural areas of South Korea. A total of 10,097 adults aged ≥65 years participated in the survey. This survey was conducted between September 14 and November 20, 2020. A trained investigator visited the participant’s home and collected data utilizing a tablet-based personal interview method. We excluded participants who responded by proxy and participants with missing data relevant to this analysis (i.e., cognitive impairment, household income, and internet use). Finally, 3,575 men and 5,064 women aged ≥65 years who used internet devices (tablets, smartphones, or computers) were included in the analysis.

### 2.3 Measurement

#### 2.3.1 Cognitive impairment

Cognitive function was assessed using the Korean version of the Mini-Mental State Examination for Dementia Screening (MMSE-DS) (30). The MMSE-DS consists of 19 items measuring general cognitive function, including time and place orientation, memory, attention, command execution, naming, copying interlocking pentagons, and judgment. The total score is calculated by summing all items, ranging from 0 to 30. Participants were classified into the categories “normal” and “impairment” using the criterion score based on gender, age, and educational level. Cognitive impairment was identified as scores that fell 1.5 standard deviations below the expected values for the age and education levels of older Korean adults (30). Mean MMSE-DS was 3 points and ranged from 1 to 5 points. This standardized test has demonstrated reliability and validity for screening cognitive impairment, including dementia.

#### 2.3.2 Social frailty

Social frailty was assessed with the instrument developed by Makizako et al. (11). This comprises five items: being alone (yes), going out less frequently compared to the previous year (yes), daily conversation with someone (no), occasional visits to friends’ homes (no), and a sense of usefulness to family and friends (no). Social frailty was categorized into three groups: robust, prefrail, and frail. For each item, a yes-or-no response was recorded. If the participant responded with two or more items indicating vulnerability, the person was classified as “frail.” If the participant responded with one item indicating vulnerability, the person was classified as “prefrail.” The participant was classified as “robust” if they exhibited no reactions to any susceptible items.

#### 2.3.3 Types of internet use

Types of internet use are generally categorized into four; information seeking, communication and social support, instrumental use, and leisure and entertainment (31). Based on previous studies, types of internet use were divided into four categories: instrumental use, interpersonal communication, entertainment, and information searching. Types of internet use was measured by asking whether the participant engaged in each of 11 types of internet activities using a Personal computer, smart phone, or tablet. Participants were asked to respond to each activity. Internet use for instrumental purposes was scored 1 for each of the following activities: shopping, internet banking, and installing applications. The same criterion was applied to other types of internet usage. Internet use for interpersonal communication included three activities; sending text messages using various social networking service applications such as Telegram and Kakao Talk; receiving text messages using such applications; and engaging in various social networking service activities such as Facebook, Instagram, and Twitter. Internet for interpersonal communication was scored as 1 if the participant engaged in any of these activities. Internet use for entertainment was coded as 1 for each of the following four activities: watching videos/movies, listening to music, playing games, and taking photos. Additionally, internet use for information searching was scored as 1 if the participant used internet for searching information such as news and daily weather. Total internet use was estimated by summing the scores for all types of internet use.

#### 2.3.4 Covariates

Age (65–69, 70–74, 75–79, 80 and over), sex, living arrangement (living alone, living with spouse, living with others), residence (urban or rural), education, equivalent annual household income, economic activity (yes, no), self-rated health (SRH) (good, fair, bad), and Instrumental Activities of Daily Living (IADL) (dependent, independent) (32) were included as covariates. Equivalent annual household income (total household income divided by the square root of the number of household members) was calculated and divided into quartiles to detect a nonlinear relationship.

### 2.4 Statistical analysis

The data were expressed as the frequencies, weighted proportions, and mean ± standard deviation (SD) of the baseline characteristics of cognitive impairment. The chi-square test and t-test were used to compare the distribution of these frequencies and means between older men and women (Table 1). We also descriptively examined the mean ± SD of types of internet use by levels of social frailty (Table 2). Logistic regression analysis was conducted to assess the impact of social frailty on cognitive impairment and the influences of the various types of internet use, while controlling for covariates (age, sex, living arrangement, residence, education, equivalent annual household income, economic activity, SRH, and IADL) (Table 3). To compare the contribution of different types of internet use, the change in odds ratios (explained fraction) before (Model 1) and after (Models 2, 3, 4, and 5) the inclusion of these types was calculated and presented using the formula: [(OR Model 1) - (OR Models 2, 3, 4 and 5) / (OR Model 1)] × 100. All statistical tests were conducted using the IBM SPSS software v.27.0 for Windows (IBM Corp., Armonk, NY, United States). The Ethics Review Board of Gachon University, with which the researchers were affiliated, approved this study [1044396-202403-HR-045-01].

**Table 1:** Characteristics of the participants by cognitive impairment.

Variables	*n* (%) or mean ± SD			*p*
	Total	Cognitively intact	Cognitively impaired	
*N=*	8,639 (100.0)	6,493 (75.2)	2,146 (24.8)	
**Gender**				
Men	3,575 (44.2)	2,654 (44.2)	921 (44.1)	0.004
Women	5,064 (55.8)	3,839 (55.8)	1,225 (55.9)	
Age	73.22 ± 6.26	72.93 ± 6.18	74.12 ± 6.39	
65–69	3,274 (35.9)	2,599 (37.7)	675 (30.2)	*p < 0*.001
70–74	2,189 (23.9)	1,641 (23.8)	548 (24.4)	
75–79	1,661 (22.4)	1,188 (21.6)	471 (24.8)	
80+	1,515 (17.8)	1,065 (16.9)	450 (20.6)	
**Education**				
No education	841 (9.0)	684 (9.8)	157 (6.4)	*p < 0*.001
Primary school	2,757 (30.1)	2,077 (30.2)	680 (29.7)	
Middle school	2,059 (23.6)	1,360 (20.7)	699 (32.4)	
High school and over	2,982 (37.3)	2,372 (39.2)	610 (31.4)	
**Living arrangement**				
Living alone	2,688 (19.9)	1,957 (19.2)	731 (21.9)	*p < 0*.001
Living with spouse only	4,489 (59.7)	3,404 (60.2)	1,085 (58.4)	
Living with others	1,462 (20.4)	1,132 (20.6)	330 (19.7)	
**Equivalent household income**				
Highst 25%	2,262 (28.8)	1,823 (30.4)	439 (24.0)	*p < 0*.001
Second 25%	2,165 (26.2)	1,645 (26.1)	520 (26.6)	
Third 25%	2,079 (22.4)	1,467 (21.2)	612 (26.2)	
Lowest 25%	2,133 (22.6)	1,558 (22.4)	702 (23.1)	
**Residency**				
Urban	6,236 (76.1)	4,747 (76.7)	1,489 (74.3)	0.08
Rural	2,403 (23.9)	1,746 (23.3)	657 (25.7)	
**Economic activity**				
Yes	3,399 (38.9)	2,672 (41.1)	727 (32.1)	0.001
No	5,240 (61.1)	3,821 (58.9)	1,419 (67.9)	
**Self-rated health (SRH)**				
Good	4,463 (51.2)	3,581 (55.0)	882 (39.6)	*p < 0*.001
Fair	2,710 (30.8)	1,934 (29.4)	776 (35.2)	
Poor	1,466 (17.9)	978 (15.6)	488 (25.2)	
**Dependence of IADL**				
Dependent	780 (9.7)	454 (7.5)	326 (16.7)	*p < 0*.001
Independent	7,859 (90.3)	6,039 (92.5)	1,820 (83.3)	
**Social frailty**				
Robust	4,274 (58.5)	3,356 (60.4)	918 (52.4)	*p < 0*.001
Prefrail	3,266 (32.2)	2,407 (31.3)	859 (34.9)	
Frail	1,099 (9.4)	730 (8.3)	369 (12.6)	
Types of Internet use (mean)	1.27 ± 1.04	1.40 ± 1.06	0.87 ± 0.87	*p < 0*.001
Instrumental use (mean)	0.10 ± 0.25	0.12 ± 0.27	0.04 ± 0.15	*p < 0*.001
Interpersonal communication (mean)	0.57 ± 0.34	0.61 ± 0.33	0.47 ± 0.34	*p < 0*.001
Entertainment (mean)	0.41 ± 0.47	0.46 ± 0.49	0.26 ± 0.40	*p < 0*.001
Information seeking (mean)	0.46 ± 0.50	0.51 ± 0.50	0.31 ± 0.46	*p* < 0.001

**Table 2:** Scores of types of internet use according to level of social frailty.

Variables	Mean ± SD					*p*
	Instrumental use	Interpersonal communication	Entertainment	Information seeking	Total	
**Social frailty**						
Roust	0.13 ± 0.29	0.65 ± 0.32	0.52 ± 0.49	0.56 ± 0.50	1.49 ± 1.07	*p < 0*.001
Prefrail	0.07 ± 0.20	0.52 ± 0.34	0.33 ± 0.44	0.38 ± 0.49	1.04 ± 0.96	*p < 0*.001
Frail	0.04 ± 0.16	0.44 ± 0.34	0.22 ± 0.38	0.27 ± 0.45	0.78 ± 0.86	*p* < 0.001

**Table 3:** Adjusted odds ratio (95% Confidence Interval) for cognitive impairment among Korean older adults and the explained fractions of types of internet use for cognitive impairment.

	Model 1	Model 2	Model 3	Model 4	Model 5
**Social frailty**					
Robust	1	1	1	1	1
Prefrail	1.32 (1.15–1.51)^***^	1.27 (1.11–1.47)^**^	1.25 (1.08–1.44)^**^	1.22 (1.07–1.41)^**^	1.28 (1.11–1.47)^***^
Frail	1.70 (1.45–2.06)^***^	1.63 (1.35–1.98)^***^	1.62 (1.34–1.97)^***^	1.55 (1.28–1.87)^***^	1.63 (1.35–1.98)^***^
**Types of internet use**					
Instrumental use		0.59 (0.53–0.66)^***^			
Interpersonal communication use			0.63 (0.59–0.67)^***^		
Entertainment				0.71 (0.68–0.75)^***^	
Information seeking					0.40 (0.35–0.46)^***^
**Explained fractions**					
Prefrail		15.6	21.9	31.2	12.5
Frail		10.0	11.4	21.4	10.0

Adjusted by age, sex, living arrangement, residence, education, equivalent annual household income, economic activity, SRH, and IADL.

†Explained Fractions in ORs for Cognitive impairment compared with the reference model was calculated using the following formula: i.e., Model 1; [(OR(Model 1) − OR(Model 2~5) /(OR(Model 1) − 1) × 100)].

*p < 0.05, **p < 0.01, ***p < 0.001.

OR, odds ratio; CI, confidence interval.

## 3 Results

Table 1 shows the descriptive statistics of the study sample. Proportions were weighted according to the sample design. Among the 8,639 participants, 75.2% were cognitively intact, and 24.8% were cognitively impaired. The mean age of older adults in the cognitively intact group (72.93 years) was higher than that of older adults in the cognitively impaired group (74.12 years). The cognitively impaired older adults were more likely to have a lower educational level and a lower household income, live alone and live in a rural area, be working and dependent, and rate their health as poorer compared to cognitively intact older adults. The prevalence of social frailty in cognitively impaired older adults (12.6%) was higher than that in cognitively intact older adults (8.3%). As for the assessment of various forms of internet use, each score of internet use was divided by the number of items assessing the types of internet use to compare the amount of each type of internet use. The mean score for interpersonal communication was the highest, while that for instrumental use was the lowest. The mean score of all types of internet use among cognitively intact older adults (Mean 1.40) was significantly higher than that in cognitively impaired older adults (Mean 0.87) (*p* < 0.001).

Table 2 compares the mean scores of all types of internet use according to the levels of social frailty. Robust older adults exhibited significantly higher mean scores across all forms of internet use, while socially frail older adults demonstrated the lowest (*p* < 0.001).

Table 3 presents the results of multiple logistic regression analysis conducted to assess the impact of social frailty on cognitive impairment and the influences of different types of internet use on that relationship, after controlling for covariates (gender, age, education, living arrangements, residency area, equivalent household income, economic activity). In Model 1, only social frailty was considered with covariates. In Models 2–5, each type of internet use was sequentially added to Model 1 to explore the effect of each type of internet use on the odds of social frailty for cognitive impairment. All the odds ratios of each type of internet use in models 2–5 were statistically significant, and the odds of levels of social frailty for cognitive impairment were significantly decreased. Among the types of internet use, the advantage of internet use for information searching was the greatest (OR 0.40, 95% CI 0.35–0.46), and that of instrumental internet use was second (OR 0.59, 95% CI 0.53–0.66), followed by internet use for interpersonal communication (OR 0.63, 95% CI 0.59–0.67) and for entertainment (OR 0.71, 95% CI 0.68–0.75). In Model 2, in which internet use for instrumental purposes was considered, the probability of cognitive impairment in the socially frail group was 1.63 (95% CI 1.35–1.98) times higher, and that in the socially prefrail group was 1.27 (95% CI 1.11–1.47) times higher than that in the robust group. It decreased by 10.0% in the socially frail group and by 15.6% in the socially prefrail group compared to the odds ratio in Model 1. In Model 2, in which interpersonal communication internet use was added, the probability of cognitive impairment in the socially frail group was 1.62 (95% CI 1.34–1.97) times higher, and that in the socially prefrail group was 1.25 (95% CI 1.08–1.44) times higher than that in the robust group. It decreased by 11.4% in the socially frail group and by 21.9% in the socially prefrail group compared to the odds ratio in Model 1. In Model 3, in which internet use for entertainment was accounted for, the probability of cognitive impairment in the socially frail group was 1.55 (95% CI 1.28–1.87) times higher, and that in the socially prefrail group was 1.22 (95% CI 1.07–1.41) times higher than that in the robust group. It decreased by 21.4% in the socially frail group and by 31.2% in socially prefrail group compared to the odds ratio in Model 1. In Model 4, in which internet use for information searching was added, the probability of cognitive impairment in the socially frail group was 1.63 (95% CI 1.35–1.98) times higher, and that in the socially prefrail group was 1.28 (95% CI 1.11–1.47) times higher than that in the robust group. It decreased by 10.0% in the socially frail group and by 12.5% in the socially prefrail group compared to the odds ratio in Model 1.

## 4 Discussion

In this study, the relationship between social frailty and cognitive impairment among community-dwelling older adults in South Korea was examined with respect to the role of internet use. As anticipated, older adults with social frailty were more likely to be cognitively impaired than those without social frailty, as evidenced by previous studies (13, 14). This study also demonstrated that all types of internet use were significantly associated with cognitive impairment and played a significant role in the relationship between social frailty and cognitive impairment. Moreover, the strength of the association between internet use and cognitive impairment, as well as the influence in the relationship between social frailty and cognitive impairment, varied depending on the type of internet use. In essence, internet use acts as a significant mechanism through which social frailty affects the cognitive function of older adults, with the specific contribution varying by the type of internet use. The differential influences of various internet usages on the relationship between social frailty and cognitive impairment are worth examining.

Among the types of internet use, the greatest benefit to cognitive function was observed with internet use for information searching, followed by use for instrumental purposes, interpersonal communication, and entertainment. This result is consistent with previous studies reporting that transitions into internet use attenuated the rate of cognitive decline, while transitions away from internet use accelerated the rate of cognitive decline (23). There are varying degrees of correlation between diverse internet use and cognitive function in older adults (31, 33, 34). Older adults may benefit from cognitive challenges when confronted with technological tasks and demands in everyday life. Among the types of internet use, information searching and instrumental use of the internet for purposes such as shopping and banking can develop the brain’s ability to access and process various information, increasing the brain’s cognitive reserve by providing cognitive stimuli. This is explained by the technological reserve hypothesis, suggesting that technology use protects cognitive capacities by providing cognitive stimulation, potentially lowering the incidence of dementia (35, 36). That is, the cognitive stimulation facilitated by internet use establishes a cognitive reserve buffer that sustains recovery from functional brain damage and promotes resilience, thereby preventing or delaying the onset of cognitive impairment (37). Furthermore, the information found on the internet may improve the health literacy of middle-aged and older adults by providing opportunities for health knowledge acquisition, improving other cognitive functions such as reasoning skills, and positively influencing healthy behaviors such as increased consumption of fruits and vegetables, less smoking, and physical activities. These behaviors, in turn, significantly reduce the risk of cognitive decline and dementia (26, 38). Meanwhile, in Yul’s study with older adults in China (34), taking part in commercial activities was the only activity that showed a negative relationship with cognitive function. However, the adverse effects decreased and were no longer significantly associated with negative cognitive function when older adults used the internet more frequently for commercial-related activities. This inconsistency with the result of this study may be attributed to the disparity in internet usage rates between China and South Korea. According to surveys in Korea, the internet usage rate among older adults aged in their 60s and those aged 70 and over was 88.8 and 38.6%, respectively, in 2018 (39), while Yul’s study reported that the rate of internet use among adults middle-aged and older was 14.9% in 2018.

As opposed to the relationship between types of internet use and cognitive impairment, internet use for entertainment exhibited the most significant influence in the association between social frailty and cognitive impairment. Interpersonal communication ranked second, followed by instrumental use and information searching. Internet use for entertainment and interpersonal communication is an intellectually stimulating social activity (34). In a study conducted by Kwon (40), which used in-depth interviews with Korean older adults aged 65 years and above, it was found that older adults perceived video-type digital contents such as YouTube, watching movies, and game-type digital contents as means to strengthen their familial bonds and facilitate communication with their grandchildren. Among the activities of internet use for entertainment, playing online games can be cognitively more stimulating than promoting social interactions. For example, playing verbal and numerical games (crosswords or Sudoku puzzles) was associated with better memory performance (41), and playing video games designed to train multitasking performance has been associated with enhanced working memory and sustained attention (42). In this study, among the internet-based entertainment activities, most participants engaged in taking photos and videos (50.6%) and watching digital contents on YouTube (34.5%), followed by listening to music (26.2%) and playing games (16.1%) [data was not shown]. Therefore, owing to the lower percentage of older adults playing online games, we presume that the association between internet use for entertainment and cognitive impairment was less significant compared to its influence on the relationship between social frailty and cognitive impairment. In conclusion, internet use for entertainment and interpersonal communication may contribute to cognitive function by reducing the influence of social frailty on cognitive impairment than by providing cognitive stimuli.

Unexpectedly, the influence of internet use between social frailty and cognitive impairment in older adults is more pronounced in older adults with prefrail status than in those with frail status. The biggest difference in the impact between prefail and frail status was when using the internet for interpersonal communication and entertainment, as opposed to instrumental use and information searching. This study classed participants as “prefrail” if they answered “vulnerable” to one of the five items—not talking to anyone, living alone, going out less frequently, feeling useless, and not seeing friends. In older adults with prefrail status, among the five items, living alone accounted for the largest percentage (42.9%), followed by going out less frequently (37.5%) [data was not shown]. We presume that internet use for amusement and interpersonal communication may compensate for reduced social connections among prefrail older adults, but not in frail older adults.

This study had several limitations. First, due to the cross-sectional study design, the causality between social frailty, cognitive impairment, and types of internet use could not be established. Second, we did not assess the frequency and duration of specific online activities, which is insufficient to reflect the wide variation in older adults’ internet usage. Finally, we did not address all the domains of cognitive function (such as complex attention or memory and processing speed), which may be important to better understand the relationship between social frailty, internet use, and cognitive function. Despite these limitations, our study used a nationally representative sample weighted by census estimates, making the findings more generalizable. Furthermore, as this investigation was conducted in Korea, where the rate of internet use by adults aged 55–74 years in South Korea (92.5%) was the eighth highest worldwide in 2022 (43), we believe that the relationship between social frailty, cognitive impairment, and types of internet use was more clearly demonstrated in this study than in studies performed in countries with limited internet access. This study is also one of the few to clearly describe the types of internet use and investigate how they influence the relationship between social frailty and cognitive impairment. These findings shed light on how different types of internet use suppress the impact of social frailty on cognitive decline.

## 5 Conclusion

Our study demonstrated that internet use has an important role in the relationship between social frailty and cognitive impairment in older adults. The influences of internet use vary depending on the types of online activity and levels of social frailty. These findings provide some evidence that various types of internet use can aid older adults with social frailty to slow down cognitive decline, indicating the potential for internet-based interventions to address cognitive impairment. Moreover, it highlights the need to consider various types of internet use and incorporate specific and effective strategies when developing non-pharmacological interventions to mitigate the impact of social frailty on cognitive decline.

## Data Availability

Publicly available datasets were analyzed in this study. This data can be found here: the data from the National Survey of Older Koreans 2020 can be downloaded via (https://www.data.go.kr) after getting approval of the researcher’s application.
